# Pulmonary and mediastinum metastasis of uterine leiomyoma: A case report

**DOI:** 10.1097/MD.0000000000018276

**Published:** 2019-12-10

**Authors:** Liqiang Huang, Gaofeng Shi, Qi Wang, Yuwei Guo, Mengdi Cong

**Affiliations:** a Department of Radiology; b Department of pathology, Hebei Medical University Fourth Hospital; c Department of pathology, Children's hospital of Hebei Province, Shijiazhuang, China.

**Keywords:** CT, mediastinum, metastatic, PET/CT, pulmonary, uterine leiomyoma

## Abstract

**Rationale:**

Pulmonary benign metastasizing leiomyoma (PBML) is rare, usually occurs in women who underwent hysterectomy during the reproductive years, and has no obvious clinical symptoms. A full understanding of the characteristics of PBML is important for its sequential treatment and prognosis.

**Patient concerns:**

In this report, a 36-year-old female patient with previous uterine leiomyoma who underwent 3 surgical resections of the uterus, bilateral fallopian tubes, and partial omentum was investigated. The physical examination revealed a tumor in the right lower lobe and mediastinum and a solid nodule in the right middle lobe.

**Diagnoses:**

Chest computed tomography (CT) confirmed a tumor in the right lower lobe and mediastinum and a solid nodule in the right middle lobe. Further positron-emission tomography computed tomography (PET-CT) with 18F-fluorodeoxyglucose (FDG) of the whole body showed mildly intense accumulation of 18F-FDG in the tumor (maximum standardized uptake value [SUV max], 2.6). A pathological examination then confirmed the presence of fibrous and vascular tissue after CT-guided percutaneous biopsy of the tumor in the right lower lobe. Additionally, surgical resection of the tumor and nodule was performed for histological analysis and immunohistochemical assays for estrogen receptor (ER) and progesterone receptor (PR).

**Interventions:**

The patient underwent complete tumor surgical resection and nodule wedge resection.

**Outcomes:**

No postoperative complications occurred. No recurrence or other signs of metastasis were found during an 18-month follow-up observation period.

**Conclusion:**

In this case, lung and mediastinal metastasis of uterine fibroids was observed. However, depending on only a postoperative histological analysis is insufficient for the diagnosis of PBML. Histological analysis combined with an evaluation of the expression levels of ER and PR is crucial for the diagnosis and treatment of PBML.

## 1 Introduction

Pulmonary benign metastasizing leiomyoma (PBML) is a very unusual disease that occasionally occurs in women who underwent a hysterectomy during the reproductive years. In 1939, Steiner first reported a case of death from pulmonary heart disease caused by multiple benign metastatic leiomyoma in the lung and mediastinum,^[1]^ and more than 150 cases of benign metastasizing leiomyoma (BML) have so far been reported in the literature,^[2]^ but there are few reports of BML metastasizing to the mediastinum. PBML is more common in women aged 34 to 55 years than in women in other age groups and has an average age of 47 years.^[3]^ The period from hysterectomy to nodule detection varies from 3 months to 20 years, with a median interval of 14.9 years.^[4]^ PBML has no clinical symptoms and is often found by physical examination or for other reasons. Immunohistochemistry plays an important role in confirming the diagnosis. Here, we present a case of uterine leiomyoma with rare metastases to the lungs and mediastinum with a fusion growth pattern. We used immunohistochemistry to detect estrogen receptor (ER) and progesterone receptor (PR) expression to confirm the diagnosis.

## 2 Case report

This study was approved by the Ethics Committee and Institutional Review Board of the Fourth Hospital of Hebei University, Shijiazhuang, China. The patient provided informed consent for publication of this case.

A 36-year-old woman was found to have a right lower lobe tumor on a computed tomography (CT) chest examination in March 2018. She had no cough, no phlegm, no blood in the phlegm and no other clinical symptoms. A routine physical examination showed no signification abnormalities. The patient previously underwent uterine leiomyoma excision in 2009 and 2012. In 2017, the patient was found to have an omental mass on a CT examination, and she then underwent resection of the uterus, the bilateral fallopian tubes and the omental tumor. The postoperative pathology of the omental tumor was leiomyoma. The tumor marker levels were normal.

**Figure 1:**
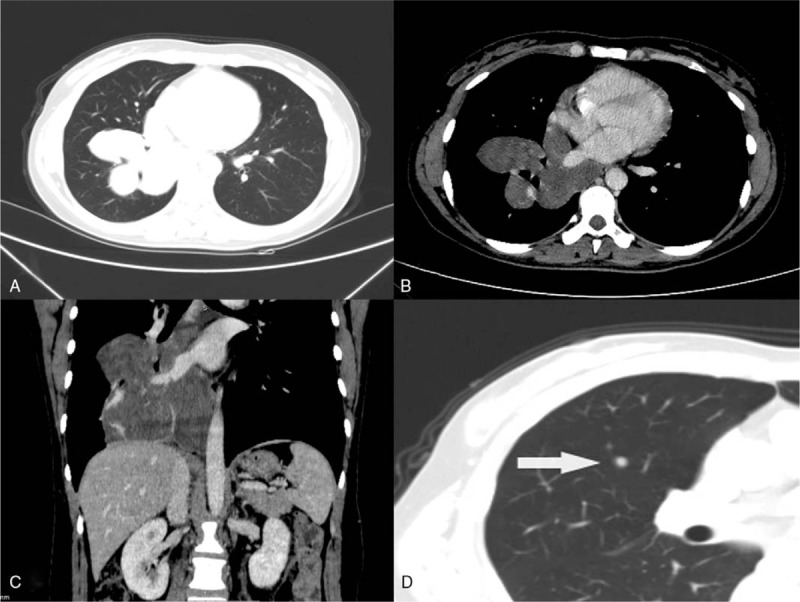
A 36-year-old woman with pulmonary benign metastasizing leiomyoma. (A, B, and C) Axial lung window, enhanced axial mediastinal window and enhanced coronal mediastinal window of computed tomography images showed a large tumor (Revise:13.2 cm × 11.1 cm × 8.9 cm) in the right lower lobe and mediastinum. (D) Axial mediastinum window of the computed tomography scan showing solid nodules (0.8 cm × 0.6 cm) in the right middle lobe (white arrows).

A chest CT examination indicated that a mass located in the right lower lobe that involved the right hilum and mediastinum showed a fusion growth pattern. The mass was characterized with an irregular form and sharp margins, and its size was approximately 13.2 cm × 11.1 cm × 8.9 cm, without cavities and calcifications (Fig. 1A). Nonuniform density was observed on a plain scan (mean CT value, 30.1 HU). In the arterial phase, which was delayed by 30 seconds, the nonvascular area was nonuniform and showed mild enhancement (mean CT value, 44.2 HU). The blood vessels and bronchi in the right lower lobe were surrounded by the mass, but no invasion was present (Fig. 1B, C). In addition, solid nodules in the right middle lobe showed a size of approximately 0.8 × 0.6 cm and had clear borders (Fig. 1D). Further positron-emission tomography computed tomography (PET-CT) with 18F-fluorodeoxyglucose (FDG) of the whole body showed a mildly intense abnormal accumulation of 18F-FDG in the tumor (maximum standardized uptake value [SUV max], 2.6) and no abnormal FDG uptake in the nodule. Pathologic results obtained following CT-guided percutaneous biopsy showed that there was fibrous and vascular tissue in the tumor, and diagnosis of benign mesenchymoma may be suggested. Finally, the patient underwent complete tumor surgical resection and nodule wedge resection.

**Figure 2:**
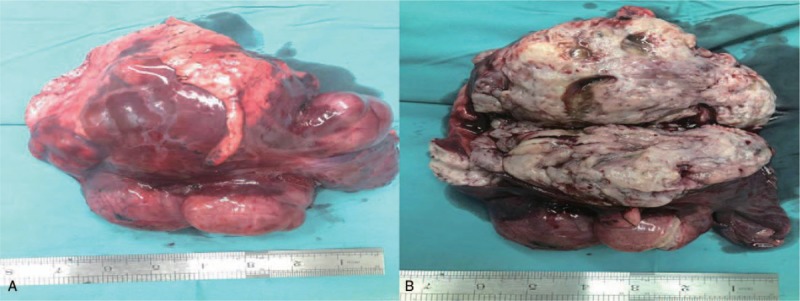
(A) Gross features of the large tumor consisting of four parts. (B) The hemisection shows that the tumor was mostly pale and tough, and the tissue structure was arranged in a braided or whirlpool shape.

**Figure 3:**
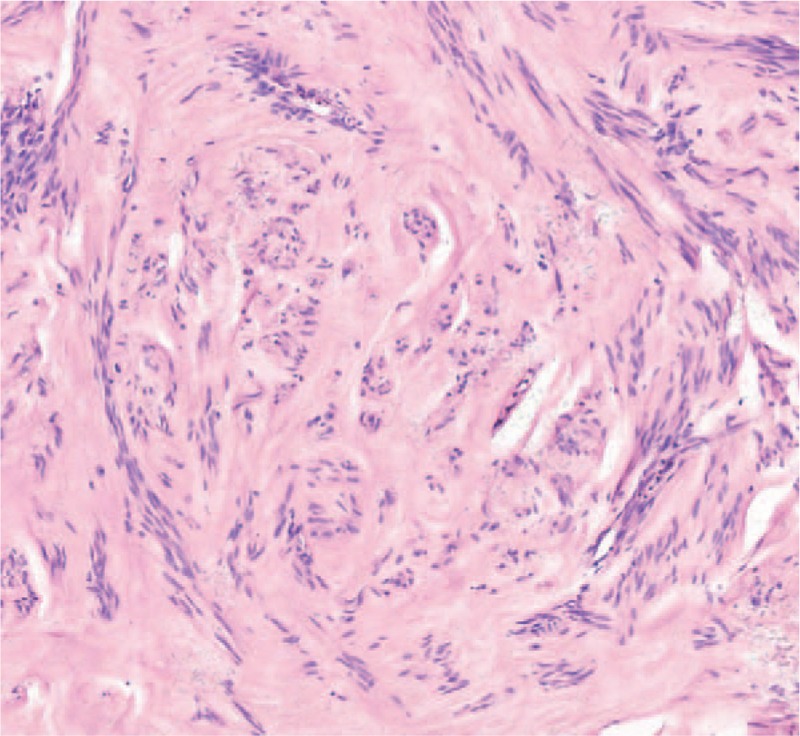
Hematoxylin-eosin staining (200×) shows that tumor cells were fusiform or ovoid, bunched together and arranged in a palisade pattern.

A gross examination indicated that the tumor consisted of 4 parts (Fig. 2A). A surface cut showed that the tumor was mostly pale and tough, its tissue structure was arranged in a braided or whirlpool shape, and the tumor had a clear boundary with normal lung tissue (Fig. 2B). Specimens of the tumor were subjected to pathological examinations. A microscopic examination of hematoxylin-eosin stained tissue demonstrated that the tumor cells were fusiform or ovoid, bunched together and arranged in a palisade pattern. Nuclear divisions were rare, and no atypia was present (Fig. 3). The immunohistochemical analysis was positive for AE1/AE3, TTF-1, CD34, CK7, EMA, SMA, Des, and calponin(+) and negative for S100(−). The proliferation index Ki-67 was positive in approximately 1% of the cells, supporting the diagnosis of leiomyoma. The solid nodule in the right middle lobe was diagnosed as leiomyoma tissue. Combined with the patient's history, the authors hypothesized that the tumor and nodule had metastasized from the uterus into the lung and mediastinum. Immunohistochemical detection of ER and PR was performed, and positive expression was revealed, resulted in a final diagnosis of PBML. The patient underwent contrast-enhanced chest-abdomen-pelvis CT examinations at the local hospital every 6 months, and no tumor recurrence or other signs of metastasis were found at the 18-month follow-up examination.

## 3 Discussion

Uterine leiomyoma is a common benign uterine tumor that occurs in women of reproductive age. Approximately 50% of women over 30 years old suffer from uterine leiomyoma.^[5]^ enign metastatic leiomyoma (BML) is very rare and is defined as a tumor with a histological appearance similar to that of uterine leiomyoma and grows outside the uterus. BML can metastasize to the lung, mediastinum, retroperitoneal area, inferior vena cava, right atrium, brain, vertebra, and skin; however, the lung is the most common site,^[6]^ and the resulting lung metastases are known as PBML. PBML usually has no clinical symptoms and is often found during a physical examination or unintentionally for other reasons. However, in some cases, patients may complain of chest pain, cough, hemoptysis and other symptoms; these are related to the location, size and number of metastatic tumors.

Awonuga et al^[2]^ retrospectively studied 150 patients and attempted to determine the origin and mechanism of BML. At present, there are 3 main theories regarding the development of PBML: 1. PBML originates from well-differentiated sarcoma of uterus.^[4,7,8]^ 2. PBML originates because of the independent development of multiple centers stimulated by abnormal hormones.^[9]^ and 3. The vascular transfer theory, which is the most widely accepted theory,^[4,10]^ states that the pathological morphology of the tumor is similar to that of uterine leiomyoma, including positive ER and PR expression, but ER and PR was uniformly negative in extrauterine leiomyomas. In addition, recent cytogenetic studies^[11–15]^ have shown that PBML has the same genetic pattern as uterine leiomyoma, and cloning studies have confirmed that benign PBML is consistent with the characteristics of uterine leiomyomas. The metastasis pattern may appear as follows: the uterine leiomyoma invades the uterine blood vessels,^[16]^ and the tumor cells form a leiomyoma plug along with the blood circulation in the pulmonary artery. They then adhere to the blood vessel wall before implanting and proliferating to form new leiomyoma nodules. Hence, hysteromyoma resection increases the possibility of metastasis. This report supports the theory of vascular metastasis.

The imaging findings^[3,17–19]^ of benign metastatic leiomyoma of the lung are similar to those of other metastatic tumors. The manifestations are multiple solid nodules in the lungs; they are rarely found as a single nodule and occasionally present as miliary nodules. The nodules vary in size, ranging from a few millimeters to a few centimeters. The nodules are usually well circumscribed and have no lobulations and cavities or calcifications. Contrast-enhanced scans usually show no enhancement or mild enhancement. Mediastinal lymph node enlargement and pleural effusion are rare. PET/CT usually shows mildly intense accumulation of 18F-FDG in the nodules.^[20]^ In this case, a mass with an atypical location invaded the right hilum and mediastinum and involved the blood vessels and bronchi in the right lower lobe. This behavior is very rare and very similar to that of a malignant tumor. PET/CT and CT-guided percutaneous biopsy are important methods for distinguishing between benign and malignant tumors in the lungs. The maximum SUV value on PET/CT for the tumor was approximately 2.6, and the CT-guided percutaneous biopsy in this report found fibrous hemangiomatous tissue. All of these findings suggested benign tumors. The CT-guided percutaneous biopsy failed to obtain a correct diagnosis, probably due to the small number of tissue samples and the absence of additional immunohistochemistry to diagnose the tumor. However, the right middle lobe nodules conformed to the imaging findings of metastases.

The diagnosis of PBML requires 5 findings^[2–4,10,16–19]^: 1. multiple or single solid nodules in the lungs with clear boundaries; 2. in general, no obvious clinical symptoms, but nonspecific symptoms, such as cough and chest pain, may be present; 3. no history of primary malignancy in other systems; 4. a history of hysteromyoma or hysteromyoma resection; and 5. histopathological morphology of the intrapulmonary leiomyoma similar to that of the uterine leiomyoma with positive ER and PR expression. In this case, the tumor showed atypical performance and was difficult to distinguish from malignant tumors. The patient had a history of two hysteromyomectomy procedures and a history of omentum leiomyoma. Omentum tumors are considered to be metastatic from the uterus. One year after surgery to treat the omentum leiomyoma, 2 masses in the right lung were unintentionally found on a chest examination, and their postoperative pathological morphology was consistent with leiomyoma. An immunohistochemical analysis revealed positive ER and PR expression, suggesting that the origin was the uterus; these findings met the diagnostic requirements for PBML.

PBML progresses slowly and has a favorable prognosis. There are currently no treatment guidelines for PBML. The specific treatment depends on the number, size, and location of the tumor, its receptor expression, the progression of the lesion, and the patient's own state. Surgical resection of metastatic tumors is an important treatment for isolated tumors. Kayser et al reported that the median postoperative survival period of 10 patients with PBML was 94 months,^[4]^ indicating that patients require close observation after surgery. No tumor recurrence or other signs of metastasis were found in a follow-up examination of our patient performed at 18 months after the operation. PBMLs are hormone-dependent tumors. Hormone therapy^[16,21–23]^ is an important adjuvant therapy and the main treatment method for tumors that cannot be surgically removed (e.g., surgical removal of the bilateral ovaries, chemical castration, long-term anti-estrogen therapy, and hormone drugs, such as progesterone and raloxifene). The aim of these methods is to reduce estrogen levels and control tumor growth. Tumors may stop growing or shrink during pregnancy or after menopause,^[19,21,24]^ but there are also cases in which metastatic tumors were found in postmenopausal women.^[25]^

Uterine leiomyoma is a common benign gynecologic tumor that can present malignant tumor behavior. Chest X-ray or CT examination can reveal solid nodules in women of reproductive age who have a history of uterine leiomyoma or surgical resection of uterine leiomyoma. We must be aware that the nodules may metastasize from the uterus. Positive ER and PR expression is an important indicator for the diagnosis of PBML that affects the choice of treatment and prognosis of patients.

## Author contributions

**Formal analysis:** Liqiang Huang.

**Investigation:** Gaofeng Shi.

**Project administration:** Gaofeng Shi.

**Resources:** Liqiang Huang, Gaofeng Shi, Yuwei Guo.

**Software:** Mengdi Cong.

**Writing – original draft:** Liqiang Huang, Yuwei Guo.

**Writing – review & editing:** Gaofeng Shi, Qi Wang.

Liqiang Huang orcid: 0000-0002-8966-414X.
